# Identification of a Phosphorylation-Dependent Nuclear Localization Motif in Interferon Regulatory Factor 2 Binding Protein 2

**DOI:** 10.1371/journal.pone.0024100

**Published:** 2011-08-26

**Authors:** Allen C. T. Teng, Naif A. M. Al-montashiri, Brian L. M. Cheng, Philip Lou, Pinar Ozmizrak, Hsiao-Huei Chen, Alexandre F. R. Stewart

**Affiliations:** 1 Department of Biochemistry, Microbiology and Immunology, University of Ottawa, Ottawa, Ontario, Canada; 2 Department of Medicine, University of Ottawa, Ottawa, Ontario, Canada; 3 University of Ottawa Heart Institute, University of Ottawa, Ottawa, Ontario, Canada; 4 Ottawa Hospital Research Institute, Ottawa, Ontario, Canada; Université Paris-Diderot, France

## Abstract

**Background:**

Interferon regulatory factor 2 binding protein 2 (IRF2BP2) is a muscle-enriched transcription factor required to activate vascular endothelial growth factor-A (VEGFA) expression in muscle. IRF2BP2 is found in the nucleus of cardiac and skeletal muscle cells. During the process of skeletal muscle differentiation, some IRF2BP2 becomes relocated to the cytoplasm, although the functional significance of this relocation and the mechanisms that control nucleocytoplasmic localization of IRF2BP2 are not yet known.

**Methodology/Principal Findings:**

Here, by fusing IRF2BP2 to green fluorescent protein and testing a series of deletion and site-directed mutagenesis constructs, we mapped the nuclear localization signal (NLS) to an evolutionarily conserved sequence ^354^ARKRKPSP^361^ in IRF2BP2. This sequence corresponds to a classical nuclear localization motif bearing positively charged arginine and lysine residues. Substitution of arginine and lysine with negatively charged aspartic acid residues blocked nuclear localization. However, these residues were not sufficient because nuclear targeting of IRF2BP2 also required phosphorylation of serine 360 (S360). Many large-scale phosphopeptide proteomic studies had reported previously that serine 360 of IRF2BP2 is phosphorylated in numerous human cell types. Alanine substitution at this site abolished IRF2BP2 nuclear localization in C_2_C_12_ myoblasts and CV1 cells. In contrast, substituting serine 360 with aspartic acid forced nuclear retention and prevented cytoplasmic redistribution in differentiated C_2_C_12_ muscle cells. As for the effects of these mutations on VEGFA promoter activity, the S360A mutation interfered with VEGFA activation, as expected. Surprisingly, the S360D mutation also interfered with VEGFA activation, suggesting that this mutation, while enforcing nuclear entry, may disrupt an essential activation function of IRF2BP2.

**Conclusions/Significance:**

Nuclear localization of IRF2BP2 depends on phosphorylation near a conserved NLS. Changes in phosphorylation status likely control nucleocytoplasmic localization of IRF2BP2 during muscle differentiation.

## Introduction

Interferon regulatory factor 2 binding protein 2 (IRF2BP2), together with the related protein IRF2BP1, were initially discovered as interacting partners to interferon regulatory factor 2 (IRF2) in a yeast two-hybrid screening assay [1]. A third homolog of IRF2BP2 called enhanced at puberty 1 (EAP1, formerly known as C14orf4) is expressed in the mediobasal hypothalamus and plays a critical function in regulating the female reproductive neuroendocrine axis [2]. All three are nuclear proteins.

Structurally, IRF2BP2 is encoded by 2 exons producing 3 alternatively spliced proteins IRF2BP2a of 587, IRF2BP2b of 561 and IRF2BP2c of 163 amino acids depending on the use of alternative donor (2a and 2b) and acceptor (2c) splice sites. IRF2BP2a and b isoforms have a Zinc-finger motif at their N-terminus, missing in the IRF2BP2c isoform, and a C3HC4 RING-finger motif at their C-terminus. The function of the Zinc-finger motif appears to enable homo- and hetero-dimerization between different members of the IRF2BP2 family[3]. The RING-finger motif from amino acids 456–587 is sufficient to interact with IRF2 [1] and also with nuclear receptor interacting factor 3 (NRIF3) [4]. IRF2BP2 was described as a co-repressor of IRF2, inhibiting the expression of interferon-responsive genes.

The tumor suppressor p53 binds to the IRF2BP2 promoter and transactivates its expression in response to actinomycin D treatments in both cervical carcinoma (HeLa) and osteosarcoma (U2OS) [5]. Increased endogenous IRF2BP2 protein levels in turn suppress the induction of apoptosis after genotoxic stress. Specifically, IRF2BP2 suppresses the transactivation activity of p53 on both Bax and p21 promoters. Anti-apoptotic activity was also ascribed to IRF2BP2 due to its modulation of a death domain in NRIF3 [3]–[4].

We identified IRF2BP2 as a cofactor of VGLL4 in a yeast two-hybrid screen [6]. VGLL4 is itself a cofactor of the TEAD transcription factors [7], that play a critical role controlling gene expression in skeletal, cardiac and smooth muscle cells [8]. We showed that transient over-expression of IRF2BP2 and TEAD1 could induce the expression of vascular endothelial growth factor-A (VEGF-A) in murine C_2_C_12_ myoblasts [6]. We also discovered that IRF2BP2 protein levels increase in response to ischemia in hindlimb and cardiac muscle. Whereas endogenous IRF2BP2 found in murine C_2_C_12_ myoblasts is nuclear, following ischemia, IRF2BP2 is mostly cytoplasmic. This discrepancy suggests a potential mechanism for modulating IRF2BP2 translocation across the nuclear membrane.

Nucleocytoplasmic shuttling is a carefully regulated process controlling the import and export of both mRNA and proteins [9]–[10]. To cross the nuclear membrane, polypeptides use different mechanisms for translocation; small proteins (<40kDa) diffuse through the membrane passively while large proteins (>40kDa) are actively transferred by the nuclear pore complexes (NPCs) on the nuclear membrane [11]. Nucleocytoplasmic transport encompasses many hierarchical steps. To initiate the process, a cargo protein heterodimerizes with karyopherin via classical nuclear localization signals (NLS; K-K/R-X-K/R, K =  lysine, R =  arginine, X =  any amino acids) [9], [12]. Next, physical binding of the guanosine-5′-diphosphate (GDP)-bound Ran molecule to the complex signals to cross the membrane. Once inside the nucleus, GTP exchange factor (GEF) facilitates the GDP to guanosine-5′-triphosphate (GTP) exchange process, leading to the release of cargo protein. One of the classical examples of a nuclear shuttled protein is the T-antigen of SV40 [13].

In this report, we used deletion and site-directed mutagenesis to localize a conserved functional NLS in IRF2BP2. In addition, we found that phosphorylation of serine residue 360 (S360) adjacent to the NLS is also involved in controlling the nuclear entry of the protein. *In silico* database mining revealed that this serine residue is phosphorylated in IRF2BP2 and IRF2BP1, and sequence comparisons revealed that this motif is conserved in all members of the IRF2BP2 family of transcription factors. Thus, our study is the first to reveal a conserved functional domain controlling nuclear import in IRF2BP2.

## Methods and Materials

### Plasmid Constructs

Human IRF2BP2a (AY278023) was first amplified by PCR with the primers 5′-GGA TCC GGC TCC TCG GACATG GCC-3′ (sense primer, BamHI) and 5′-TCT AGA GTC TCT CTC TTT TTT CAC TTT-3′ (antisense primer, XbaI) and was then subcloned into TOPO vectors (Invitrogen, Carlsbad, CA). To generate pEGFP-IRF2BP2a, full-length IRF2BP2a was released from TOPO-IRF2BP2a plasmid by BamHI digestion and then subcloned in pEGFP-C1 (Clontech, San Jose, CA). A summary of the GFP-IRF2BP2 fusion constructs is shown in Fig. 6. To generate the GFP-TEAD4 expression construct, TEAD4 was first amplified by PCR with the following primers: 5′- GAA TTC TGG AGC CTT GGA GGG CAC GGC - 3′ (EcoRI) and 5′- GGA TCC CCG AGT CTC TCA TTC TTT CAC CAG -3′ (BamHI) and subcloned into pEGFP-C1 vector between EcoRI and BamHI sites. For generating mutant constructs, the IRF2BP2 mutants – 355 GAC GAC GAC GAC 358 (aspartic acids), TCT 360 TAT (S360A), and TCT 360 GAT (S360D) - flanked between the endogenous PstI and NcoI in IRF2BP2 were ordered from Integrated DNA Technologies (San Diego, CA) as pIDTSMART-IRF2BP2 and were subcloned in pEGFP-IRF2BP2a (Cloning details will be provided upon inquiry). Similarly, a pEGFP-IRF2BP2b construct was generated by swapping a PstI/NcoI fragment of IRF2BP2b into the pEGFP-IRF2BP2a clone. All plasmid constructs were purified by CsCl-banding and their reading frames were verified by sequencing. DsRed-Rab11 WT (Addgene plasmid 12679) and RFP-LAMP1 (Addgene plasmid 1817) expression vectors were obtained from Addgene (Cambridge, MA). Expression vectors for wild type IRF2BP2, S360A and S360D mutants were generated by subcloning a full-length EcoR1 fragment from pEGFP-IRF2BP2 into the pXJ40-CMVHA vector. The mouse VEGFA promoter construct was described previously [6].

### Tissue culture, transient transfections and luciferase assays

C_2_C_12_ myoblasts and African green monkey kidney (CV1) cells were obtained from American Type Culture Collection (Rockville, MD). Cells were maintained in high glucose DMEM with 20% FBS (or 10% FBS for CV1), 100 U/ml penicillin, 100 ug/ml streptomycin. Lipofectamine 2000 (Invitrogen, Carlsbad, CA) and GenJet Reagent (Cat # SL100489- C_2_C_12_, SignaGen Laboratories, Ijamsville, MD) were used to transfect CV1 and C_2_C_12_ myoblasts, respectively, according to the manufacturer's instructions. Luciferase assays were conducted as described previously [6] and luciferase activities were expressed as mean fold ± SEM relative to the empty pXJ40CMV vector. Differences between fold luciferase activities were compared by Student's t-test and considered significant at p<0.05.

### Protein kinase inhibitors

The protein kinase A (PKA) inhibitor H89 (working concentration: 20 µM, Cat. #: B1427), the inhibitor of phosphoinositide 3-kinases LY294002 (10 µM, L9908), and the MEK1/MEK2 inhibitor U0126 (10 µM, #U120) were from Sigma-Aldrich (Oakville, ON., Canada). The calcium/calmodulin-dependent protein kinase II (CaMKII) inhibitor CK-59 (100 µM, #208922) and the ribosomal S6 kinase (RSK) inhibitor SL0101 (10 µM, #559285) were from Calbiochem (Gibbstown, NJ). The cyclin dependent kinase inhibitor olomoucine (10 µM, #V2372) was from Promega (Madison, WI).

### Immunofluorescence

The rabbit polyclonal anti-IRF2BP2 antibody was described previously [6]. Rabbit polyclonal PRDX3 antibody (ab50300) was purchased from Abcam Inc., (Cambridge, MA). Alexa488-conjugated goat anti-mouse and Alexa594-conjugated goat anti-rabbit antibodies were from Santa Cruz Biotechnology Inc., (Santa Cruz, CA). 48-hours post transfection, cells were washed twice with 1x phosphate-buffered saline (PBS; 3.2 mM Na_2_HPO_4_, 0.5 mM KH_2_PO_4_, 1.3 mM KCl, 135 mM NaCl, pH 7.4), fixed in ice-cold methanol at −20°C for 10 minutes, washed thrice with 1x PBS, permeabilized by 0.5% Triton-X 100, and washed thrice with 1x PBS. Cells were then counter-stained with 4′,6-diamidino-2-pheylindole (DAPI, Sigma), mounted on glass slides, and visualized on a Zeiss M1 microscope.

## Results

**Figure 1:**
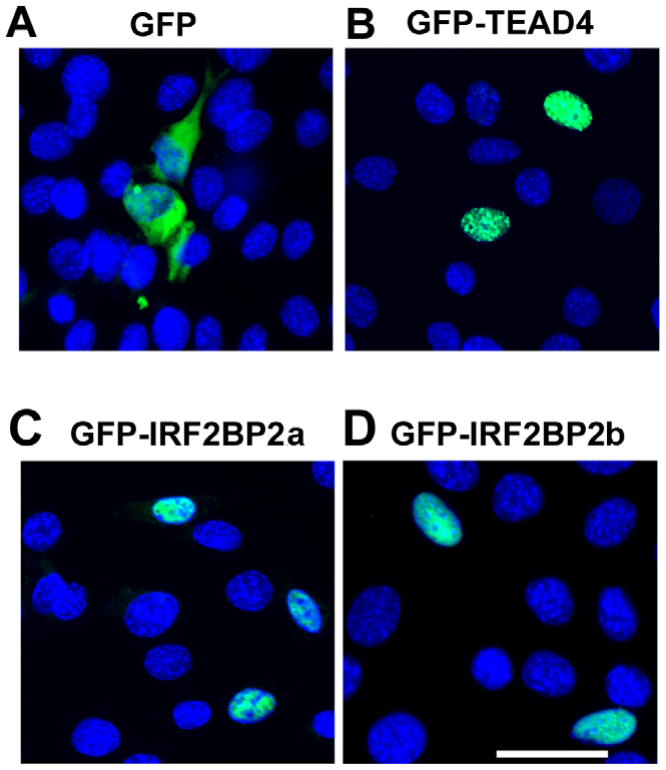
Green fluorescent protein (GFP) full-length IRF2BP2 fusion constructs are targeted to the nucleus. A. GFP alone was localized in the cytoplasm of C_2_C_12_ myosblasts. B. A GFP fusion construct with the nuclear-targeted transcription factor TEAD4 was nuclear localized in C_2_C_12_ myoblasts. C. GFP-IRF2BP2a was localized to the nucleus of C_2_C_12_ myoblasts. D. GFP-IRF2BP2b was also nuclear in C_2_C_12_ myoblasts. Nuclei were revealed by DAPI in blue. Scale bar, 50 µm.

IRF2BP2b is a nuclear protein in mouse C_2_C_12_ myoblasts that becomes redistributed to the cytoplasm during muscle differentiation [6]. The nuclear targeting sequence has not been identified in IRF2BP2 so we generated green fluorescent protein (GFP)-IRF2BP2 fusion constructs to enable the identification of sequences necessary and sufficient for this process. The GFP protein is localized to the cytoplasm of C_2_C_12_ myoblasts whereas a control construct fusing GFP to the TEAD4 transcription factor is localized to the nucleus of C_2_C_12_ myoblasts (Fig. 1A). The GFP-IRF2BP2a and GFP-IRF2BP2b fusion proteins were detected in the nucleus of C_2_C_12_ myoblasts (Fig. 1C,D). Since the both isoforms localized to the nucleus, we chose IRF2BP2a for subsequent studies.

**Figure 2:**
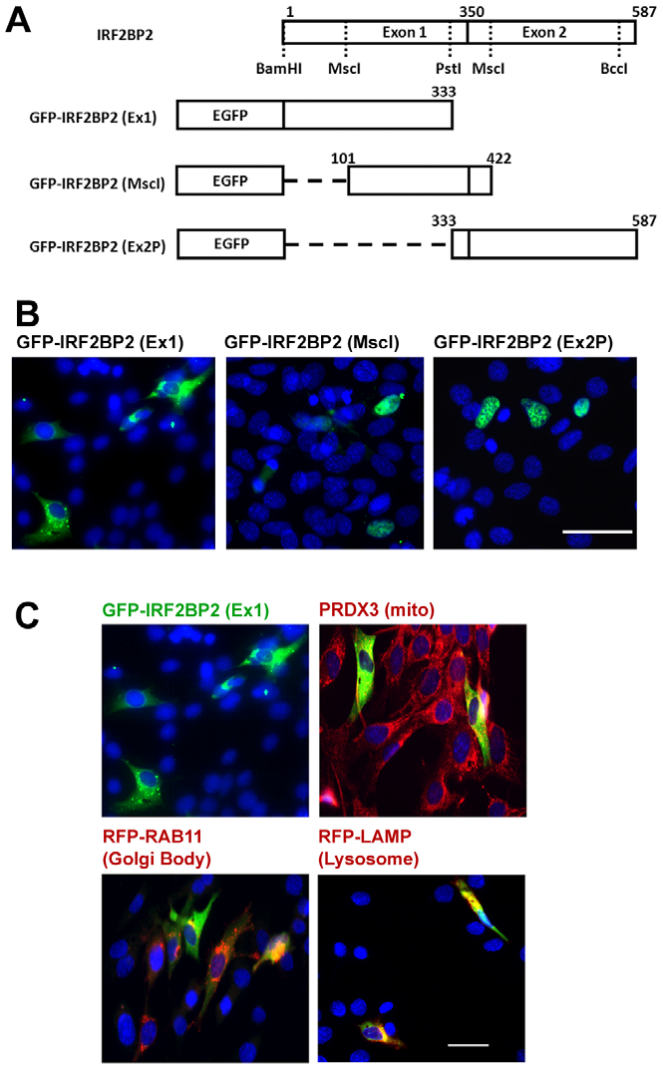
Nuclear localization signal is contained within the C-terminal half of IRF2BP2. A. Diagram of GFP fusion IRF2BP2 deletion contructs used to localize the NLS. B. The N-terminal fragment, GFP-IRF2BP2(Ex1) is cytoplasmic, whereas the middle, GFP-IRF2BP2(MscI), and C-terminal, GFP-IRF2BP2(Ex2P), fragments are nuclear in C_2_C_12_ myoblasts. C. The N-terminal fragment is targeted to the lysosome in C_2_C_12_ myoblasts. Mitochondrial-specific peroxiredoxin 3 (PRDX3) and Golgi body-sepcific Ras-related protein 11 (Rab11) did not, whereas the lysosome-associated membrane protein (LAMP) did co-localize with GFP-IRF2BP2(Ex1). Scale bars, 50 µm.

Using convenient restriction sites, we found that the N-terminal sequence of IRF2BP2 encoded by exon 1 (amino acids 1-333) was not imported into the nucleus, whereas a fragment encoded largely by exon 2 (amino acids 333–587) was localized in the nucleus (Fig. 2). A fragment containing amino acids 101–422 was also localized in the nucleus, identifying a nuclear targeting sequence between amino acids 333 and 422. The GFP-IRF2BP2(Ex1) fusion protein was found in cytoplasmic speckles (Fig. 2B,C). To determine the cellular localization of this fragment, we tested for colocalization with a mitochondrial protein (peroxiredoxin 3, PRDX3), a lysosome associated membrane protein (LAMP) and a Golgi body targeted protein Ras-related protein 11 (Rab11) using specific antibodies. We found that the N-terminal fragment of IRF2BP2 is targeted to the lysosome, and is likely degraded (Fig. 2C).

**Figure 3:**
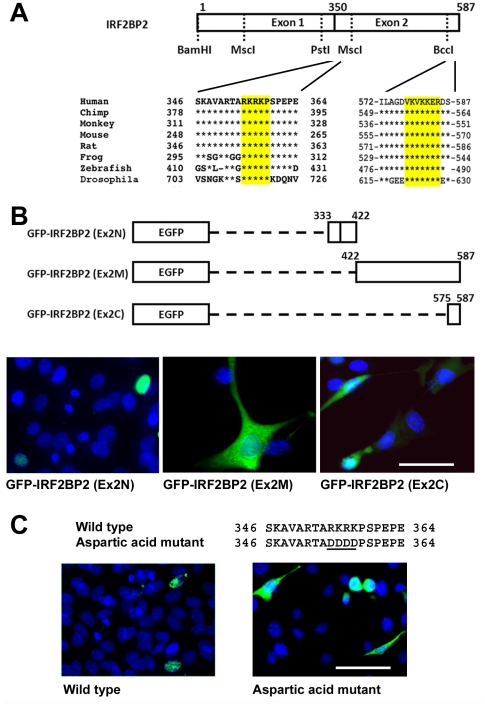
Identification of the NLS within the C-terminal half of IRF2BP2. A. Two putative NLS sequences were identified in conserved regions of IRF2BP2. Core arginine (R) and lysine (K) sequences are highlighted in yellow. B. GFP fusion constructs with the amino acid positions indicated were tested in C_2_C_12_ myoblasts to reveal that the sequence between amino acid 333 and 422 contains the functional NLS of IRF2BP2. C. Site-directed mutagenesis converting the R and K to aspartic acid (D) residues prevented nuclear localization. Scale bar, 50 µm.

Given that the C-terminal fragment of IRF2BP2 is targeted to the nucleus, we sought to identify the minimal nuclear localization sequences (NLS) within this fragment. Bioinformatic analyses (Fig. 3A) suggested two putative NLS in conserved regions of IRF2BP2, one at the N-terminus of exon 2 encoded sequence we called Ex2N, ^357^ARKRKP^364^ and the other and the C-terminus we called Ex2C, ^579^KVKKERD^587^. To evaluate whether these sites are functional, the second exon was further divided into three parts, namely 333-422, 422–587, and 575–587, fused with GFP, and then transiently overexpressed in C_2_C_12_ myoblasts. Here, the 333–422 fragment was found in the nucleus (Fig. 3B), but the C-terminal Ex2C 575–587 fragment or the 422–587 fragment did not. Thus, the Ex2C sequence is not an NLS, nor is a cryptic NLS present in the sequences from amino acids 422 to 587. To authenticate the NLS at Ex.2N, positively charged amino acids were replaced by negatively charged aspartic acids (DDDD) using site-directed mutagenesis (Fig. 3C). When expressed in C_2_C_12_ myoblasts, the aspartic acid mutant remained in the cytoplasm, confirming that the Ex2N NLS in IRF2BP2 is functional (Fig. 3C).

**Figure 4:**
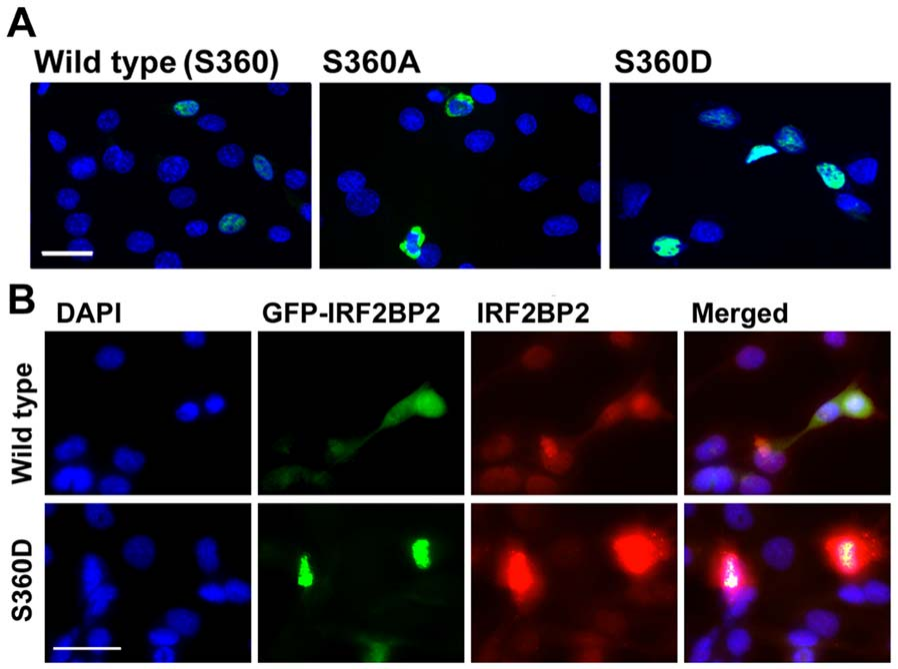
Phosphorylation of serine 360 controls nuclear localization of IRF2BP2. A. Site-directed mutagenesis of serine 360 to alanine (S360A) blocked nuclear localization of IRF2BP2 whereas aspartic acid substitution (S360D) did not differ from wild type and was nuclear localized in C_2_C_12_ myoblasts. B. After 72 hours in differentiation medium, the endogenous IRF2BP2 (revealed with IRF2BP2 antibody), as well as GFP-IRF2BP2 (wild type, green), are localized in both the cytoplasm and nucleus of C_2_C_12_ myotubes. The S360D mutant was exclusively found in the nucleus in C_2_C_12_ myotubes.

A survey of phosphopeptide databases generated from large scale proteomic studies that employed mass spectrometry revealed that IRF2BP2 is phosphorylated at serine 360 (S360), two amino acids downstream of the newly identified Ex2N NLS, in many human cell types (Table 1). To test whether phosphorylation of S360 affects nuclear import of IRF2BP2, we mutagenized the serine to either an alanine (S360A) that cannot be phosphorylated or to an aspartic acid (S360D), that carries a negative charge and mimics phosphorylation. The GFP-IRF2BP2 S360A mutant was localized in the cytoplasm of C_2_C_12_ myoblasts. To rule out that the S360A mutant created a cryptic nuclear export signal, leptomycin B (10 nM) was used to block CRM1-dependent nuclear export, but it did not cause nuclear retention of the GFP-IRF2BP2 S360A mutant (Fig. S1). Thus, cytosolic localization of the GFP-IRF2BP2 S360A mutant likely results from failure to import rather than a CRM1-dependent nuclear export. In contrast, the GFP-IRF2BP2 S360D mutant was located in the nucleus (Fig. 4A). During skeletal muscle differentiation of C_2_C_12_ cells, we reported previously that IRF2BP2 is partially relocated to the cytoplasm [6]. Thus, we asked whether the S360D mutant would enforce nuclear retention of IRF2BP2 in C_2_C_12_ cells 72 hours after the induction of muscle differentiation. Both the endogenous IRF2BP2 and the GFP fusion protein bearing full-length wild type IRF2BP2 were localized in both the cytoplasm and nucleus, whereas the S360D mutant was exclusively nuclear (Fig. 4B).

**Table 1:** Serine 360 of IRF2BP2 is phosphorylated in multiple human cell types.

Human Cell line/tissues	Identified peptides	Reference
HeLa (cervical carcinoma)	RKPpSPEPEGEVGPPK	[26]
NCI-H1299 (non-small cell lung cancer)	KPpSPEPEGEVGPPK	[28]
HeLa (cervical carcinoma)	KRKRpSPEPEGEVGPPK	[29]
Embryonic stem cells (male)	KPpSPEPEGEVGPPK	[30]
MV4-11 (myeloid)	RKPpSPEPEGEVGPPK	[31]
Jurkat (T lymphocytes)	VARTARKRKPpSPEPEGEVGPP	[27]
Hepatocellular carcinoma	RKPpSPEPEGEVGPPK	[32]
Leukocyte-blood	RKPpSPEPEGEVGPPK	[33]

Fragments analyzed by mass spectrometry were identified by mining online datasets.

**Figure 5:**
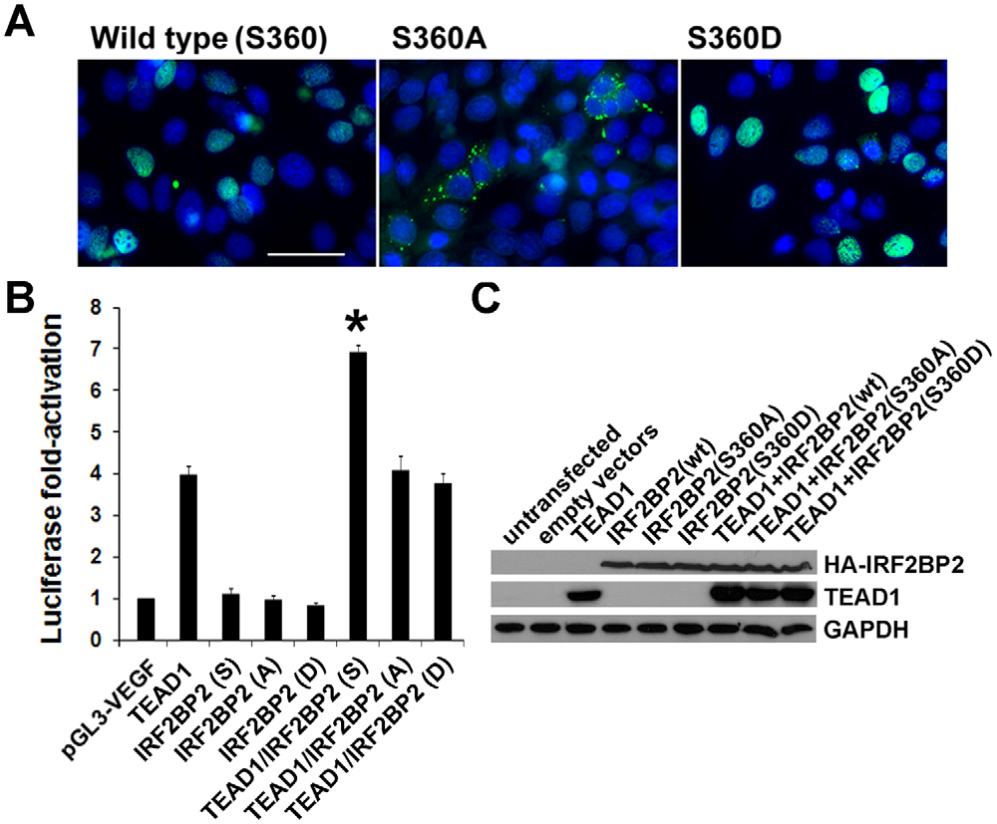
Despite forced nuclear retention the S360D mutant did not produce a dominantly active form of IRF2BP2. A. The S360A mutant of IRF2BP2 was cytoplasmic and the S360D mutant was nuclear in CV1 cells. Scale bar, 50 µm. B. The wild type IRF2BP2 augmented TEAD1-dependent activation of the mouse VEGFA promoter in CV1 cells (asterisk, p<0.05). The S360A and the S360D both failed to co-activate VEGFA when co-expressed with TEAD1. N = 3 experiments. C. Immunoblot analysis of protein lysates from transfected CV1 cells revealed similar levels of wild type and mutant IRF2BP2 proteins.

When co-expressed with the transcription factor TEAD1, we reported previously that IRF2BP2 strongly co-activates a mouse VEGFA promoter in African green monkey kidney CV1 cells, where endogenous IRF2BP2 levels are low[6]. We also observed nuclear exclusion of the S360A mutant of IRF2BP2 and nuclear retention of the S360D mutant in CV1 cells (Fig. 5A). We next tested whether nuclear exclusion of IRF2BP2 would block and whether forced nuclear retention of IRF2BP2 would enhance mouse VEGF promoter activity in the presence of TEAD1. Compared to wildtype IRF2BP2 co-expressed with TEAD1, not only did the alanine mutant fail to co-activate the VEGFA promoter, as expected, but surprisingly the aspartic acid mutant also failed to co-activate the VEGFA promoter in CV1 cells (Fig. 5B). We confirmed that TEAD1 and the wild type and the mutant IRF2BP2 proteins were expressed at comparable levels and were of the expected size by immunoblot analysis of transfected CV1 cells (Fig. 5C). Thus, although the aspartic acid substitution of serine 360 forces nuclear retention of IRF1BP2, it also appears to disrupt the co-activation function of IRF2BP2.

Currently, we do not know which kinase is responsible for the phosphorylation of S360. From a list of candidate kinases includes protein kinase A (PKA, consensus sequence R-R/K-X-S/T), kinase B (PKB), kinase C (PKC), kinase G (PKG), ribosomal S6 kinase (RSK), extracellular signal-regulated kianses 1/2 (ERK1/2, X-X-S/T-P), calmodulin-dependent protein kinase II (CaMKII, R-X-X-S/T), and Cdc2 (S/T-P-X-R/L), we tested various inhibitors against these kinases to determine whether they would cause cytoplasmic retention of IRF2BP2. However, in no case was IRF2BP2 excluded from the nucleus of C_2_C_12_ myoblasts 24 hours post-treatment (Fig. S2).

**Figure 6:**
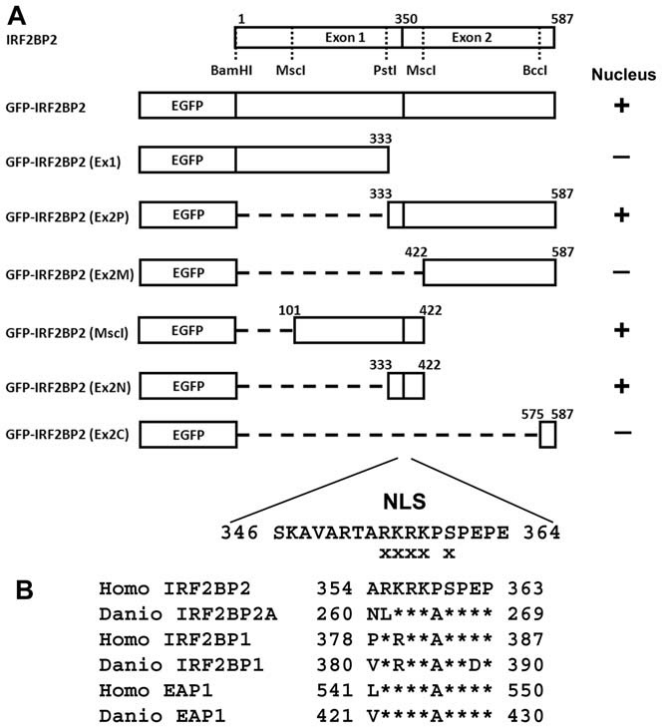
Identification of the phosphorylation-dependent NLS of IRF2BP2. A. Diagram of deletion and mutagenesis constructs used to identify the phosphorylation-dependent NLS. Fusion proteins that are targeted to the nucleus are identified by a +, whereas those that are cytoplasmic are identified by -. Exes (x) represent mutagenized residues that abolish nuclear localization. B. Sequence conservation of the NLS. In all 3 paralogous genes, IRF2BP2, IRF2BP1 and EAP1 in humans (Homo sapiens) and zebrafish (Danio rerio), the NLS and the phosphorylation target sequences are conserved. Asterisks represent identical sequence.

The diagram shown in Fig. 6A summarizes our finding of a single phosphorylation-modulated NLS in IRF2BP2. This sequence is conserved across the different paralogous homologs in both humans and zebrafish (Fig. 6B).

## Discussion

Nuclear import of IRF2BP2 depends not only on a classical nuclear localization sequence, but also on phosphorylation of serine 360. Although our studies have not identified the protein kinase responsible for this phosphorylation event, the cytoplasmic localization of IRF2BP2 in differentiating skeletal muscle cells suggests that this protein kinase might become down-regulated or inactivated. Preventing nuclear entry of IRF2BP2 by mutating S360A was sufficient to render the protein inactive to regulate VEGFA promoter activity. Surprisingly, forced nuclear localization did not make IRF2BP2 a better transactivator of VEGFA. This result indicates that nuclear localization is not sufficient to ensure transcriptional activity of IRF2BP2 and that the S360D mutation might affect the interaction of IRF2BP2 with a putative transcription cofactor or affect a transactivation function of IRF2BP2.

Nuclear trafficking is a fundamental and essential mechanism to modulate import and export of RNA and proteins across the nuclear membranes within eukaryotes. This process depends heavily on thousands of protein complexes on the nuclear envelope and the import/export signals on cargo molecules. The classic NLS, composed of a cluster of arginine and lysine residues, has been extensively studied. X-ray crystallography shows that the positive charges from lysine and arginine enhance the binding affinity to negatively charged armadillo pockets of karyopherin-α [14]. Karyopherin-α serves as an adaptor between a cargo protein and karyopherin-β. Nevertheless, other studies have shown that karyopherin-α could directly interact with a cargo protein [15]. Our study showed that one of two putative sequences was a functional NLS required for IRF2BP2 for nuclear entry.

Post-translational modifications are important for modulating protein structures or functions for downstream actions in response to environmental cues. One of frequently encountered examples includes phosphorylation [17]–[20]. Database mining revealed that IRF2BP2 is phosphorylated at several serine residues in addition to serine 360 (data not shown). Serine 360 is located two amino acids downstream from the NLS (Fig. 6B) and our site-directed mutagenesis studies are consistent with phosphorylation at this site being necessary for nuclear entry, because blocking this process by mutagenesis led to perinuclear accumulation of the mutant protein (S360A), even in the presence of the NLS. A possible explanation is that phosphorylation of the serine residue is likely to alter IRF2BP2 protein structure, strengthening the stability of IRF2BP2-karyopherin complexes for crossing the nuclear membrane. Many examples for phosphorylation-dependent nuclear entry exist in eukaryotes, including import of extracellular signal-regulated kinase-1/2 (ERK1/2) [21], histone deacetylase 4 (HDAC4) [22], forkhead box M1 (FOXM1) [23], and adenomatous polyposis cell (APC) protein [24]. However, we were not able to identify the kinase that phosphorylates S360. This might be due to phosphorylation of S360 by more than one kinase. For example, low-density lipoprotein (LDL)-receptor-related protein 6 (LRP6) can be phosphorylated by both glycogen synthase kinase 3 (GSK3) and casein kinase I (CKI) [25].

The discovery of a single classical NLS in IRF2BP2 may help to predict the NLS in both IRF2BP1 and EAP1. The results show a striking similarity in sequence conservation, with both paralogs having a conserved NLS and a conserved phosphorylation target in an adjacent serine (Fig. 6B). Mass-spectrometry confirms that both S384 of IRF2BP1 [26] and S547 of EAP1 [27] are phosphorylated in vivo. It remains to be formally proven whether both NLS signals and the serine residues provide similar functions as those observed in this study for IRF2BP2. It is worth noting that the locations of these sequences are not in currently known functional domains. This is consistent with previous studies showing that an NLS is most often away from other functional motifs to avoid interference. Our study provides important insight into the position of the nuclear targeting signals in the IRF2BP2 family of transcription factors.

## Supporting Information

Figure S1 **Blocking CRM1 nuclear export did not cause retention of the S360A mutant of IRF2BP2**. C_2_C_12_ myoblasts transfected with GFP-IRF2BP2 (S360A) were treated with vehicle (1 µl 70% methanol in 1 ml medium, Control) or leptomycin B (10 nM, LMB). Scale bar, 10 µm.
(TIF)

Figure S2 **Various protein kinase inhibitors failed to block nuclear import of endogenous IRF2BP2.** C_2_C12 myoblasts were treated with vehicle (DMSO) or inhibitors of protein kinases for 24 hours. Scale bar, 50 µm. See Methods and Materials for descriptions and concentrations of protein kinase inhibitors.
(TIF)
