# Supplementary figures and images for: Identification of a Phosphorylation-Dependent Nuclear Localization Motif in Interferon Regulatory Factor 2 Binding Protein 2
Source: PLoS One. 2011 Aug 26;6(8):e24100. doi: 10.1371/journal.pone.0024100 (PMC3162591; doi:10.1371/journal.pone.0024100)

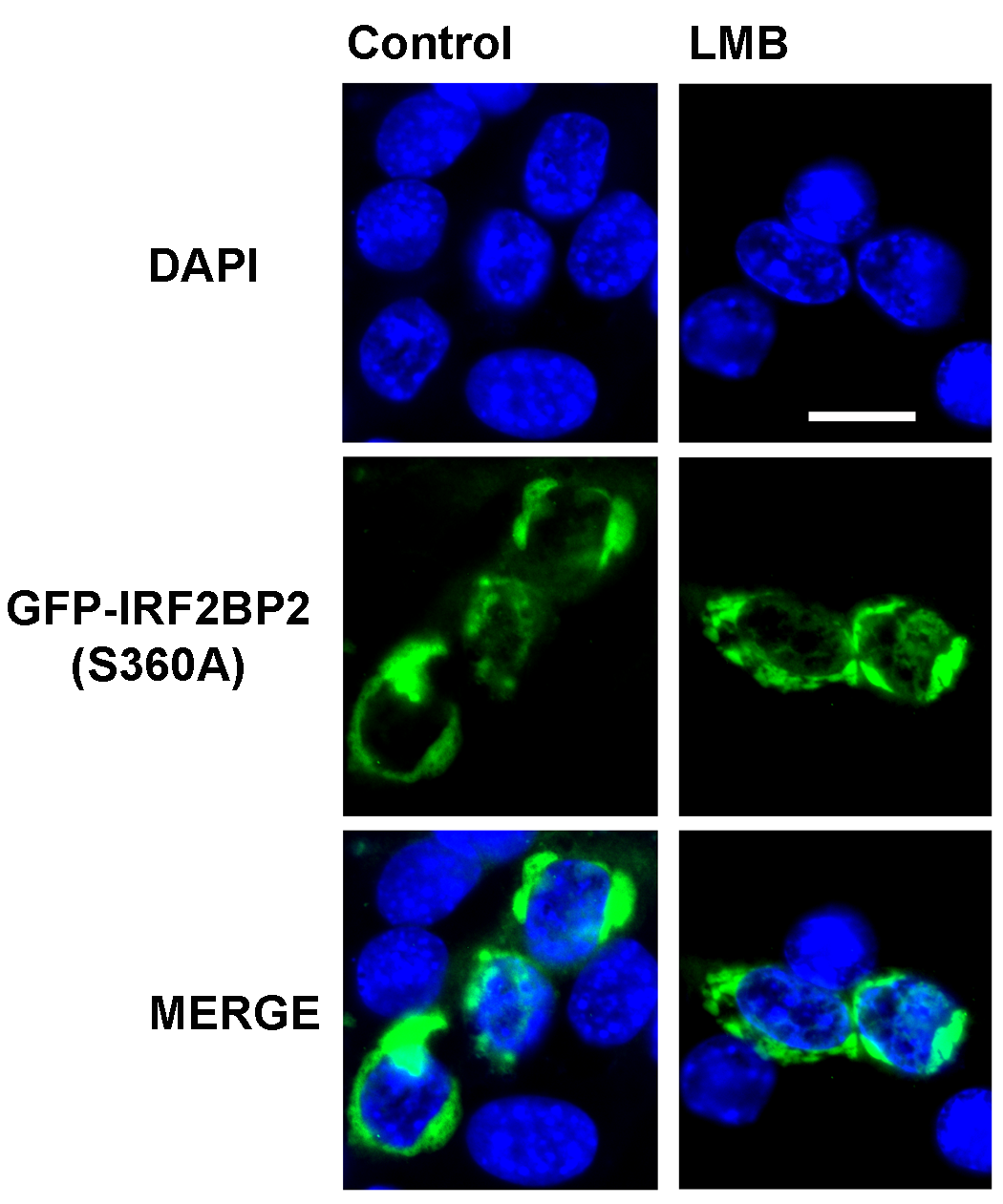

Supplement: Figure S1 — Blocking CRM1 nuclear export did not cause retention of the S360A mutant of IRF2BP2. C2C12 myoblasts transfected with GFP-IRF2BP2 (S360A) were treated with vehicle (1 µl 70% methanol in 1 ml medium, Control) or leptomycin B (10 nM, LMB). Scale bar, 10 µm. (TIF) [file pone.0024100.s001.tif]

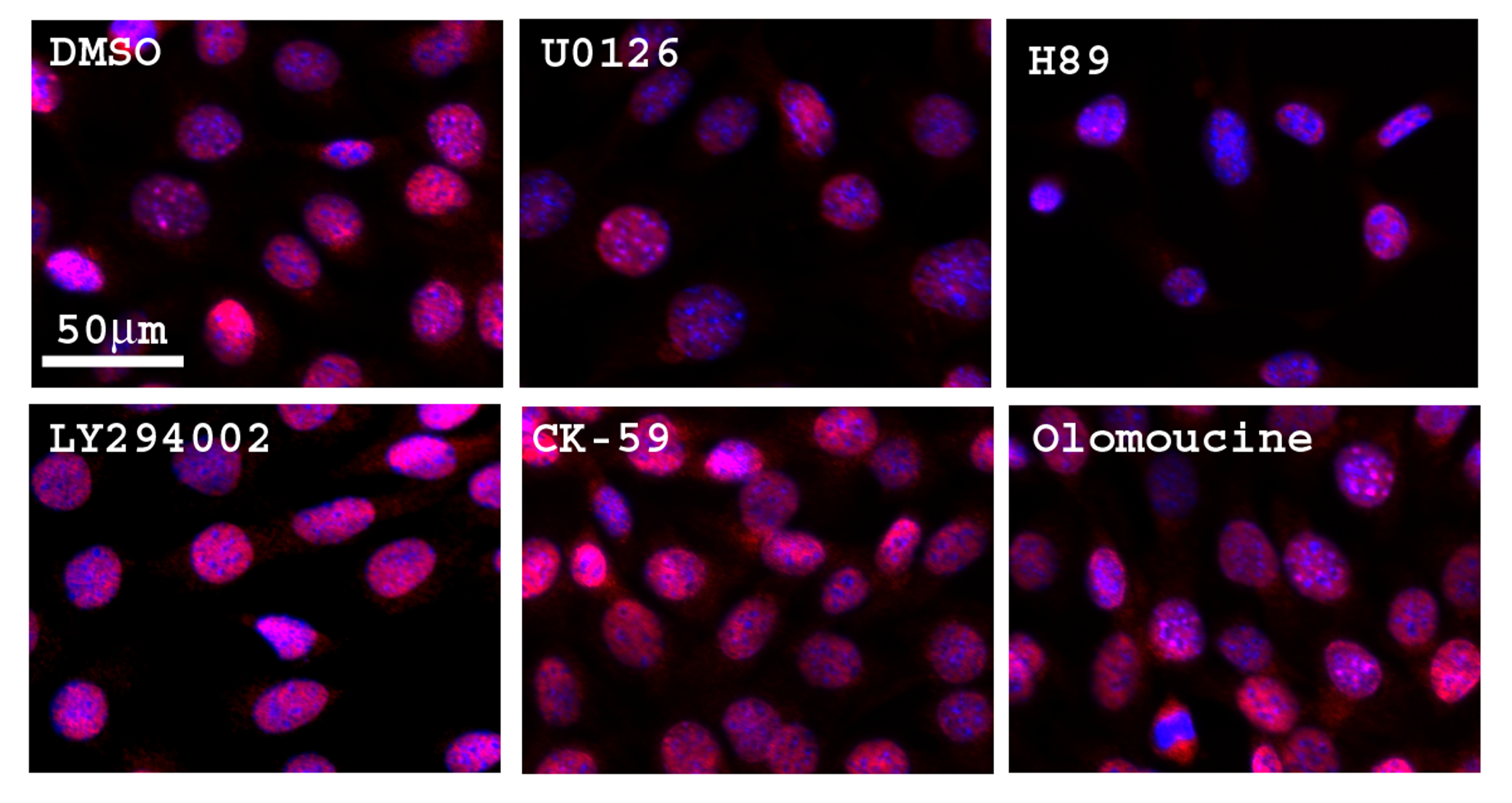

Supplement: Figure S2 — Various protein kinase inhibitors failed to block nuclear import of endogenous IRF2BP2. C2C12 myoblasts were treated with vehicle (DMSO) or inhibitors of protein kinases for 24 hours. Scale bar, 50 µm. See Methods and Materials for descriptions and concentrations of protein kinase inhibitors. (TIF) [file pone.0024100.s002.tif]
